# Maximum gain enhancement in wireless power transfer using anisotropic metamaterials

**DOI:** 10.1038/s41598-023-32415-9

**Published:** 2023-05-12

**Authors:** William Carter Harris, David S. Ricketts

**Affiliations:** ECE Department, North Carolina State University, Raleigh, 27606 USA

**Keywords:** Electrical and electronic engineering, Metamaterials

## Abstract

We present an analysis for metamaterial (MM) enhanced wireless power transfer (WPT) that includes new results revealing the impact of magnetostatic surface waves and their degradation of WPT efficiency. Our analysis shows that the commonly used fixed loss model used by previous works leads to the incorrect conclusion regarding the highest efficeincy MM configuration. Specifically, we show that the “perfect lens” configuration provides lower WPT efficiency enhancement in comparison to many other MM configurations and operating conditions. To understand why, we introduce a model for quantifying loss in MM-enhanced WPT and introduce a new figure of merit on efficiency enhancement, $G_{\rho }$. Using both simulation and experimental prototypes, we show that while the “perfect-lens” MM achieves a field enhancement of four times the other configurations considered, its internal loss due to magnetostatic waves significantly reduces its efficiency-enhancement. Surprisingly, all the MM configurations analyzed other than the “perfect-lens” achieved higher efficiency enhancement in simulation and in experiment than the perfect lens.

## Introduction

Non-radiating, magnetoquasistatic wireless power transfer (WPT) is an attractive means to efficiently transfer power over short to medium distances. One of the key parameters for high-efficiency power transfer is the coupling coefficient between the source and load. One promising proposed enhancement technique is to use a near-field metamaterial (MM) placed between the source and load. An initial demonstration^1^ showed the first experimental evidence that a MM could increase power transfer by demonstrating a 35% increase in $S_{21}$ using two MMs. Several subsequent works^2–4^ have demonstrated further improvements in WPT using MMs. They showed enhancement qualitatively through the increased brightness of a light bulb and quantitatively using an enhancement of coupling via $S_{21}$, where $S_{21}$ is more than doubled. These initial works utilized a MM configuration that has isotropic permeability, i.e, the same in all directions. In particular, they operated at a permeability of -1, which was shown to create a “perfect lens^5^,” or “super lens^4^,” that focuses sub-wavelength magnetic fields. Subsequent works^6–10^ have looked at MM-enhanced WPT, many of which have adopted one type of anisotropic MM with resonators all coaxial with the WPT transmitter and receiver (called a Z-MM in this paper). This structure is simpler than the perfect lens and has led to further improved results using MM enhanced WPT.

While demonstrating the viability of, and improvements in, MM enhanced WPT, these previous works did not present an analytical and experimental framework for optimization of WPT using generalized MM. These works principally built a MM and placed it between two coils and then measured $S_{21}$ for various positions until an optimal $S_{21}$ was measured. While an excellent demonstration, they did not provide the insight necessary for general MM WPT analysis. In particular for multiple anisotropic MM structures that were not “perfect lens” or “superlens”^4^. The simpler co-axial coil anisotropic configuration while effective, is not the only MM that provides enhanced WPT. In fact, our results show that a different anisotropic MM than used by many researchers provides a higher efficiency. Moreover, the negative effect of magnetostatic waves on WPT efficiency has not been systematically investigated for isotropic and anisotropic MMs.

In this work, we present a complete methodology for analysis, characterization and implementation of MM enhanced WPT using a simulation and experimental framework. Our analysis shows that the loss induced by magnetostatic surface waves mitigates much of the benefit of field enhancement using a “perfect-lens” structure. As a result, the induced loss in MM due to magnetstatic waves becomes a dominant metric in understanding and realizing high-efficiency MM enahnaced WPT. While many works^6–10^ have shifted to non-perfect lens configurations, they have not provided a general analysis to analyze and compare different anisotropic MM for the maximum gain (efficiency) enhancement.

**Figure 1:**
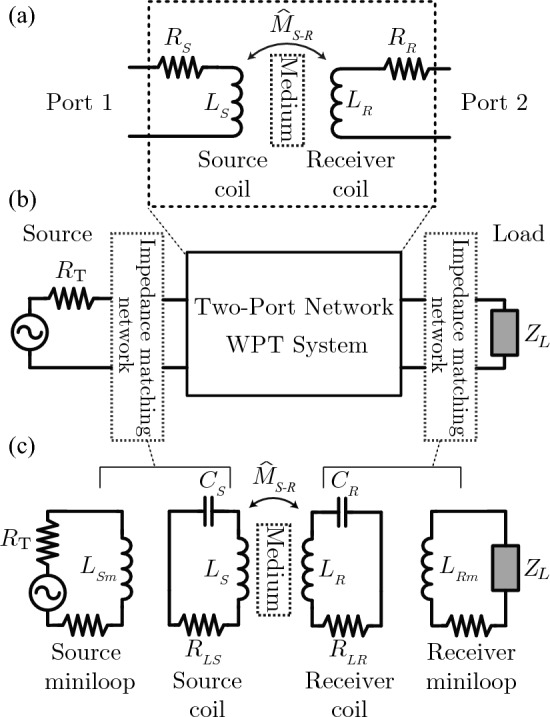
(**a**) Circuit for a two-coil WPT system with arbitrary medium between source and receiver. (**b**) Two-port representation of a WPT network, with impedance matching two-port networks used to maximize power transfer from source to load. (**c**) 4-coil circuit representation of mini-loop impedance matching technique for WPT. This is also known as 4-coil WPT and magnetic resonance coupling, due to the the configuration of the coils as resonators. These terms all describe the same mechanism: impedance matching the WPT coils to the source and load.

The WPT system used in our analysis is shown in Fig. 1a. A two-coil setup is used, where a single transmitter coil is coupled to a receiver with some mutual inductance $\hat{M}_{S-R}$. We seek to deliver maximum power from the source, whose internal resistance is $R_T$, to a load with impedance, $Z_L$. We previously showed^11^ that this can be done by impedance matching the load and source to the WPT system. A mini-loop impedance match is used to achieve this^12^, which is equivalent to strongly coupled magnetic resonance^11,13^. The circuit that describes this configuration is shown in Fig. 1c.

Figure 1b also shows that the two-coil setup may be viewed as a generic two-port network, with *Z*- or *S*-parameters. This is a particularly attractive approach as it does not depend on whether the system has a material between source and receiver or not; the two-port parameters completely characterize the system. In particular, impedance, or *Z*-parameters, are an intuitive set of two-port parameters that allow for insight into the behavior of the WPT system. If the *Z*-parameters can be measured, then the system circuit parameters can be readily deduced. For any *two*-coil WPT system (MM present or not), the effective two-port *Z*-parameters can be defined in terms of the effective coil (source and receiver) resistances $R_{S}$ & $R_{R}$, inductances $L_{S}$ & $L_{R}$, and mutual inductance $\hat{M}_{S-R}$. This corresponds to writing the general Z-parameters of the circuit in Fig 1a:

1 $$\begin{aligned} Z = \begin{bmatrix} R_{S} + j \omega L_{S} &{} j \omega \hat{M}_{S-R}\\ j \omega \hat{M}_{S-R} &{} R_{R} +j \omega L_{R} \\ \end{bmatrix} \end{aligned}$$

Note that for coils in air only, $\hat{M}_{S-R}$ is a purely real number. When a MM is used the form of the *Z*-parameters remains the same, however $\hat{M}_{S-R}$ becomes complex (the hat indicates a complex quantity). The complex mutual inductance captures the effects of the MM. By defining $\hat{M}_{S-R}=\hat{k} \sqrt{L_S L_R}$, the complex mutual inductance also results in a complex coupling coefficient, $\hat{k}$.

Once the two-port representation of the system is known, it is straightforward to compute the maximum possible efficiency of the two-coil system^14^. The maximum possibly efficiency can be computed from explicit *Z*-parameters or using the *Q*-factors of the resonant coils and the (possibly complex) coupling coefficient $\hat{k}$ between source and receiver. The maximum achievable efficiency is:

2 $$\begin{aligned} \chi= & {} {\frac{ \left| Z_{21}\right| ^{2}}{{\text {Re}}\{ Z_{11}\}{\text {Re}} \{Z_{22}\}- {\text {Re}} \{Z_{21}^2\}}} \text{ or } \end{aligned}$$

3 $$\begin{aligned} \chi= & {} {\frac{ \left| k \right| ^{2}Q_{{S}}Q_{{R}}}{1- {\text {Im}} \{ k \} ^{2}Q_{{S}}Q_{{R}}}} \end{aligned}$$

4 $$\begin{aligned} G_{max}= & {} \frac{\chi }{ ( 1+ \sqrt{1+\chi } )^2} \end{aligned}$$

where $Q_{S}$ and $Q_{R}$ are the effective quality factors of the source and receiver resonant coils, including induced losses due to the MM, and $Q_{S,R}=\omega L_{S,R}/(R_{S,R}+R_{Eq.-MM})$. The MM affects the self inductance of the coils since it has a bearing on how much flux is coupled through each loop ($\mu _r \ne 1$ everywhere around coils), and likewise the MM induces added loss due to the loss in the coupled resonant coils of the MM^15^.

In addition to ohmic losses in MM electrical components, an important and significant loss mechanism also exists through the excitation of Magneto-quasistatic waves (MSW)^16–18^. MSW can occur in a layered structure where the center material has a different permeability than the two outer layers, e.g. $\mu _r$ and $\mu _r'$, Fig. 4. In the context of this work, the MM has a permeability $\mu _r$ that is different than the surrounding air ($\mu _r'=1$). Two types of magneto-quasistatic waves can occur: magnetostatic volume waves (MSV), Fig. 4a,b, when $\mu _{xx,yy}$ and $\mu _{zz}$ have different signs and magnetostatic surface waves (MSW), Fig. 4c,d, when $\mu _{xx,yy}$ and $\mu _{zz}$ are the same sign^19^.

**Figure 2:**
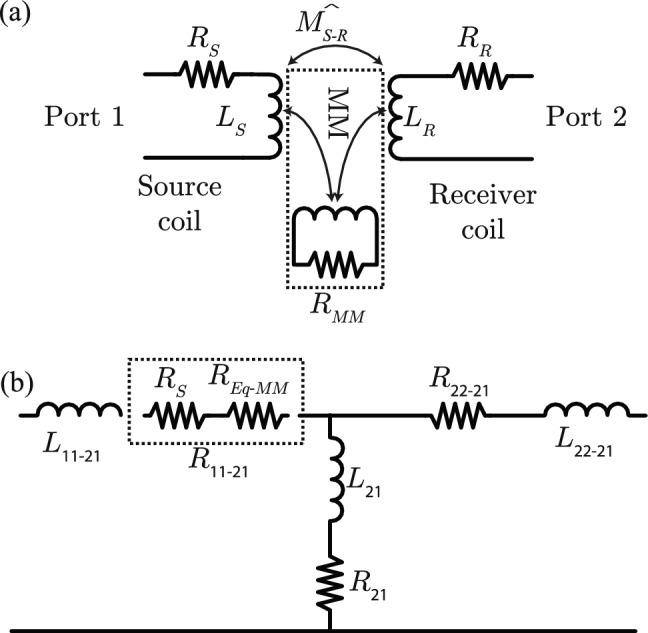
(**a**) proposed model of MM. Loss is modeled as an effective 3rd loop coupled to both source and receiver. (**b**) A T-model of the coupled inductors. The equivalent loss through the magnetostatic waves (MSW) is captured in $R_{Eq-MM}$.

To model the general loss of the MM, we propose the model shown in Fig. 2a, which is similar to models used to incorporate eddy current loss in substrates of integrated inductors^20^ and WPT^21^. The loss induced by the inclusion of the MM is modeled as a coupled dissipative element, $R_{MM}$, which is used to model the loss in the MM due to component loss, e.g. resistance of MM coils, as well as the energy lost due to magnetostatic waves (MSW). Figure 2b shows the equivalent T-model of two coupled inductors with the equivalent dissipation of the MM represented as $R_{Eq-MM}$. In the T-model, the series element is calculated as $Z_{11}-Z_{21}$. We separate the real and imaginary parts and focus on the real part for loss. As can be seen, $Z_{11}$ is determined by both the added loss of the MM as well as the real part of the increase.

This additional loss can be also viewed through $\chi$ in $G_{max}$. In examining $\chi$, one sees that the numerator is the coupling and the denominator resembles the dissipative element, $R_{11}-R_{21}$. We shall show that the denominator is qualitatively similar to the dissipative element and we will use *D* to represent the denominator and as a metric of loss. The denominator, *D*, is defined as

5 $$\begin{aligned} D={1- {\text {Im}} \{ k \} ^{2}Q_{{S}}Q_{{R}}}={\text {Re}} \{Z_{11}\}{\text {Re}} \{Z_{22}\}- {\text {Re}} \{Z_{21}^2\} \end{aligned}$$

and $\chi$ can be rewritten as:

6 $$\begin{aligned} \chi =\frac{Z_{21}}{D}=\frac{1}{\omega }\frac{\hat{M}}{D}. \end{aligned}$$

It is clear that maximization of $\chi$ and with it $G_{max}$ is dependent on both $\hat{M}$ and *D*.

Previous works on analytical analysis of MM WPT have focused mainly on $\hat{M}$ or $Z_{21}$, where they compared the increase in coupling to that of the air (no MM) case. They defined the parameter

7 $$\begin{aligned} \rho =\frac{Z_{21-MM}}{Z_{21-air}}, \end{aligned}$$

which measures the increased coupling. There is also an increase in coupled loss due to the MM, that is not captured in $\rho$. We therefore use a new term, introduced in^22^, $G_\rho =G_{M-MM}/G_{M-air}$, which is the *efficiency enhancement* of the system and better captures the improvement of any MM (or other) enhanced WPT as it considers both the field enhancement, $\rho$, and increased dissipation through *D*. Using $G_\rho$, we investigated MM enhanced WPT and found that the induced losses due to MSW result in a dominating factor that shifts the optimal MM from a theoretical one of the perfect lens to an anisotropic MM operated at $\mu _r\ne -1$.

## Results

We analysized two simulation geometries and one experimental apparatus. The two simulations differ in their boundary conditions, i.e. infinite versus finite size. The infinite simulation model allows analysis without the effects of reflections of MSW from the MM edges. The finite simulation model includes reflections and other size effects, most notably Fabry-Perot resonances of MSW in the MM. The finite simulation also matches the dimensions of the experimental apparatus and is used for comparison.

### Metamaterial simulation of infinite disc—constant loss vs. matched loss

We first investigated a MM enhanced WPT system with a circular MM slab of infinite radius with three permeability configurations: an XY MM ($\mu _x=\mu _y\ne 1$and $\mu _z=1$), a Z MM ($\mu _x=\mu _y=1$, and $\mu _z\ne 1$) and an XYZ MM ($\mu _x=\mu _y=\mu _z\ne 1$). For each configuration we simulated $G_{\rho }$ versus peremability, where the MM real part of the permeability is varied from $-6< {\text {Re}}\{\mu \} < 6$. This allows easy analysis of the behavior versus material properties. Previous works^4^ simplified the analysis by assuming a constant imaginary component of the permeability - or constant loss. In the first set of simulations, we also used a fixed value ${\text {Im}}\{\mu \}=0.2j$ for all real permeabilities. This represents an optimistic loss for a realizable structure.

**Figure 3:**
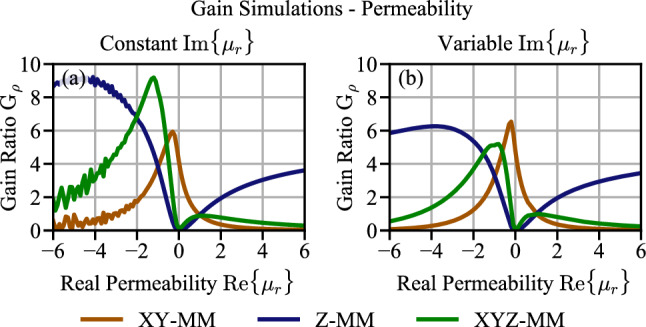
A comparison of the simulated gain for the XY, Z, and XYZ MMs with (**a**) constant ${\text {Im}}\{ \mu _r \}$ and (**b**) matched loss: variable ${\text {Im}}\{ \mu _r \}$.

Figure 3a shows the resulting gain enhancement for this fixed loss. The highest $G_{\rho }$ occurs in the isotropic MM, when it is operated at a $\mu _r$ at or near $-1$. This is exactly where the perfect lens condition occurs^22^. Alternatively, one might consider a Z configuration operating at a $\mu _r$ between $-4$ and $-6$. Note that by the definition of $\mu _r$, the field magnitude of magnetic field in the Z direction should be the same at $\mu _r=-4$ or $\mu _r=4$, however the efficiency enhancement is not. We attribute this to the magnetostatic waves that are excited for negative permeability, which further increase the field in the coils^19^.

A constant loss provides an easy metric to investigate the desired permeability and anisotropy. However a MM constructed as an array of resonant coils will have a frequency dependent complex permeability^23^. To include this frequency dependent loss, we simulated $G_{\rho }$ versus $\mu _r$, however varied the imaginary part of $\mu _r$. The imaginary part was matched to the real part obtained in our experimental setup (see “Methods”), such that for each real part in the abscissas we used the imaginary component associated with that real part from experiments. We will refer to this set of complex permeabilities as matched loss. Figure 3b shows the efficiency enhancement, $G_{\rho }$ versus $\mu _r$ for matched loss. Notably, the relative magnitudes (peak) change when the matched loss is used instead of the fixed value of ${\text {Im}}\{\mu \}$. Notably, the XYZ, which had the highest gain enhancement for the fixed loss now has the lowest (peak) gain.

### Metamaterial simulation of a finite disc and magnetostatic waves

In order to understand the origin of this shift in gain enhancement (and surprising change from the perfect lens to a non-perfect lens as the optimal case for WPT enhancement), we examined the loss mechanisms due to magnetostatic waves. We used a similar simulation setup as before, however we used a finite disc in order to better match our experimental apparatus. The parameters are plotted versus frequency instead of ${\text {Re}} \mu$. This does not change the analysis as it still uses the matches loss peremabilities, however it aligns the simulation results with experimental results, which we present subsequently.

**Figure 4:**
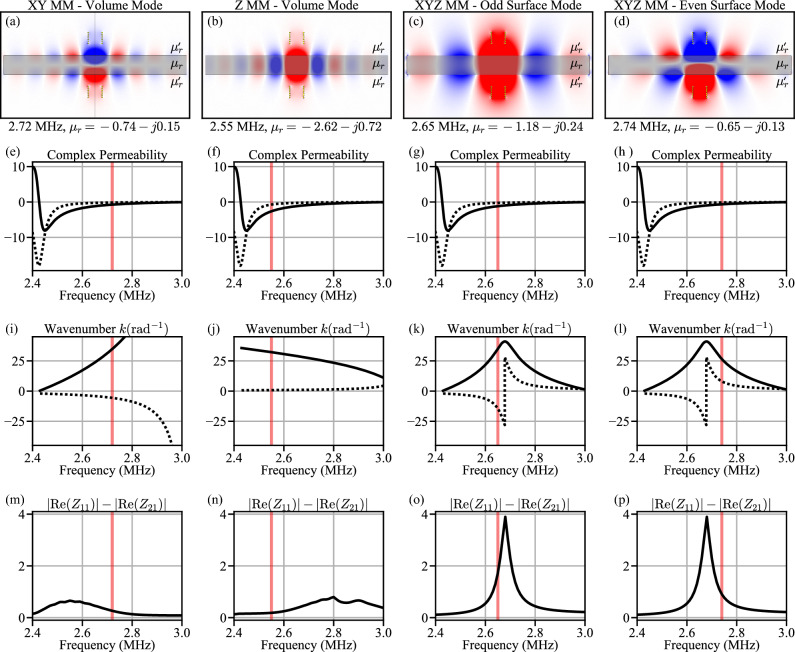
(**a**–**d**) magnetostatic waves for XY, Z, and XYZ (two cases) MM. Shown is magnetic Flux $B_z$. (**e**–**h**) complex permeability used for calculations of each MM. (**i**, **j**) complex wavenumber calculated from (9) and (**k**–**l**) from (10). The dashed is the real component and the dotted is the imaginary component. (**m**–**p**) dissipative term calculated from Z-parameters from simulated MM, Fig. 6b. The red vertical line shows the frequency for the field plots in (**a**–**d**).

Figure 4a–d show a representative excited magnetostatic wave mode in each MM configuration for single frequency and thus single complex permeabilities extracted from our experimental prototype, Fig. 4e–h. In Fig. 4 we switch our abscissa variable to frequency, so that the simulation can be compared to experimental measurements. This change does not affect the results as the same pairing of real and imaginary $\mu _r$ values is used plotting versus $\mu _r$ as is used for plotting versus frequency. The modes in Fig. 4a–d are at the frequency (and complex permeability) shown by the vertical red lines for each configuration in Fig. 4e–p. For the XY-M and Z-MM (Eq. 10) we plot only the first order mode of the magnetostatic volume mode wave where $m=0$. For the XYZ-MM (Eq. 9), we plot both solutions, the even and odd surface mode designated by the ± in Eq. (9).

Figure 4i–l plot the real and imaginary parts of *k*, where the imaginary component represents the loss of the magnetostatic waves. Figure 4m–p plot the series dissipative term, $|R_{11}|-|R_{21}|$ from the *T*-model. This loss was calculated from the real part of the *Z*-parameters extracted from a 2D axisymmetric simulation (see “Methods”), which included the finite geometry of the MM. One can see that the peak loss of the XYZ-MM occurs in the series dissipative term exactly where the imaginary part of *k* is also at its highest. Thus, the dissipative term matches well with the simulated loss of the XYZ MM and its imaginary part of the wavenumber. The loss in the XY-MM and Z-MM also correlate with the propagation constant, however due to the finite size of the MM, the supported MSW are limited to the lower (XY-MM) and higher (Z-MM) frequencies and thus, Fig. 4m,n the peak loss does not occur with the highest values of the imaginary part of the wavenumber, Fig. 4i,j, since the region where the imaginary part is highest, no MSWs occur and thus lower loss is seen in (m) and (n) in these regions. Said differently, loss occurs when there is a MSW and at the region with the highest loss for which there is a MSW.

### Metamaterial experimental results

**Figure 5:**
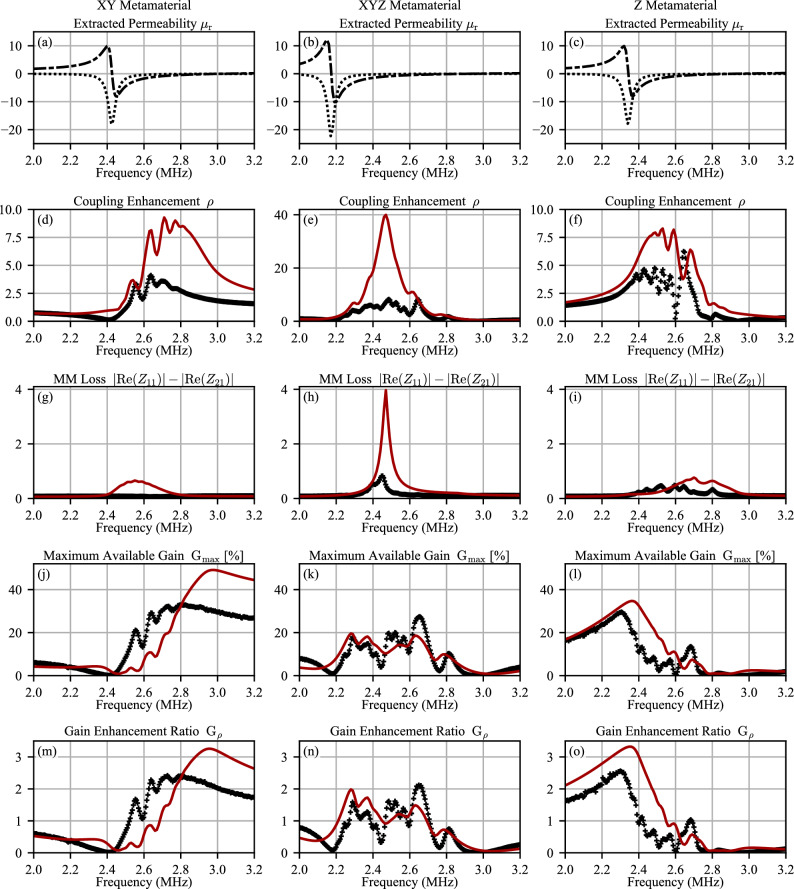
Simulation and experimental results for the Z, XY and XYZ MM. (**a**–**c**) Show the extracted permeabilities form the experimental prototype. These were used to do a matched simulation. (**d**–**i**) Show the calculated parameters $\rho$ and *D*. From these $G_{max}$ was calculated, (**j**–**l**). Finally (**m**–**o**) show the gain enhancement ratio, $G_{\rho }$.

Figure 5 plots the key calculated parameters for the simulated case above and the experimentally measured cases of air and the three MM configurations. The loss and gain enhancement are encompassed by these parameters. The experimentally-extracted complex permeability for the XY-, Z-, and XYZ-MMs are shown in Fig. 5a–c; note that all of the plots in Fig. 5 are a function of system frequency. These three permeability plots drive the behavior of the MM, which affects the frequency response of the other MM- and WPT-parameters and consequently the suitability of each MM configuration for WPT applications. Figure 5d–o shows the results for each parameter: $\rho$, *D*, $G_{max}$, and $G_\rho$. The simulated results are shown as a solid red trace and the measured results in black crosses. Figure 5d–f show the coupling enhancement $\rho$ of the system with each MM in place. A value of $\rho =1$ indicates that the coupling between the WPT coils is equivalent to the system with no MM in position (free space only between coils).

Figure 5g–i shows the series dissipative term $|R_{11}|-|R_{21}|$, for each MM configuration. A larger value indicates that the MM experiences greater losses at that frequency, including the loss of the magnetostatic mode present in the MM. This parameter indicates that the XYZ-MM experiences significant losses when compared to the other two configurations. Additionally, the peaks shown in Fig. 5f,i occur at the same frequency, which means the coupling enhancement that the XYZ-MM offers is mitigated in part by the presence of elevated losses.

The parameter $G_{max}$, the maximum available gain, offers a more complete picture of the performance of the WPT-MM system; Equations (4) and (6) show how the enhancement of mutual coupling ($Z_{21}$) and the diminishing effects represented by *D* (related to $|R_{11}|-|R_{21}|$) are both taken into consideration. Finally, $G_\rho$ is calculated to provide a direct comparison of the WPT system gain with and without the MM. A value of $G_\rho =1$ indicates that the system gain is equivalent to the gain of the system with only free space between the WPT coils.

## Discussion

Figure 4a–d reveal several insights about the MMs considered. The first is that the loss (dotted line) of the XY-MM and Z-MM are much lower than the XYZ-MM in the range of 2.4–2.8 MHz, where there is enhanced coupling, $\rho$. The peak loss of the XYZ-MM occurs at the frequency where $\mu _r\approx -1$ (see Fig. 5h], or the perfect lens condition. This aids in explaining why the XYZ-MM efficiency in Fig. 3 reduced significantly from an assumed constant loss to the matched loss simulation; the loss is much higher at the perfect lens condition than any other area. In addition the effects of the MSWs can be seen as they are generated in the regions of high $\rho$, thus they are an important, and previously not considered, part of the analysis of enhanced wireless power transfer.

**Figure 6:**
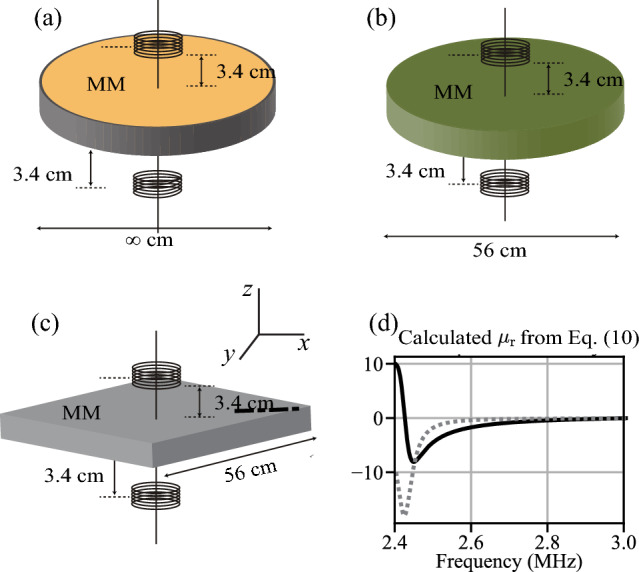
(**a**) Simulation setup for an infinite MM. It uses an axisymmetric disc with boundary conditions that emulate an infinite structure. (**b**) The simulations for the experimental comparison. We use an axisymmetric disc with finite diameter equal to the width of our experimental prototype. There is a discontinuity in the boundary conditions from the disc to the surrounding air as is also the case with the experimental prototype. (**c**) Experimental setup for comparison to the simulation setup. Coil configuration and distance are the same as simulation, however the MM is rectangular. (**d**) Calculated permeability (real—black, and imaginary—gray) vs. frequency using Eq. (8) and $Q=50$, $\omega _o=2\pi \cdot 2.425MHz$ and $F=0.35$.

Figure 5d–f plot the simulated and measured $\rho$, which represents the enhancement of transmission (S21) compared to air. Peak enhancement is between 2.4 and 2.8 MHz. The variability is due to different permeabilities for each configuration and their geometry (i.e. how they enhance the coupling). The XYZ-MM has a vertical axis that is scaled four times the the other configurations, such that its enhancement is four times the scale of the XY- and Z-MM. This is consistent with previous works^4^. Figure 5g–i plot the loss term defined in Fig. 2. This is the key parameter that has not been previously widely considered. It shows that the loss term for the XYZ-MM is more than four times that of the other configurations and occurs exactly at the point of the perfect lens. As discussed above and shown in Fig. 4a–d, the isotropic MM supports two wave modes. At the point where ${\text {Re}}\{\mu \}\approx -1$, both waves have significant loss, resulting in significant loss in the MM. Owing to this disproportionate increase in loss (compared to enhancement $\rho$), the maximum possible gain, $G_{Max}$ is flattened in the region of ${\text {Re}}\{\mu _r\}\approx -1$. Likewise, the XY-MM $G_{Max}$ is reduced in the region 2.4–2.8 MHz, where the MSW loss is significant and the Z-MM $G_{Max}$ is reduced in the region 2.5–2.8 MHz for the same reason.

The XYZ-MM, which has the greatest coupling enhancement (4x the other two configurations) but also the greatest loss, can reach 1.7 times the gain of the WPT system without a MM, except for one isolated point where it reaches 2.1. Figure 5k shows the maximum efficiency ($G_{Max}$) versus frequency. This peak $G_{\rho }$ in the XYZ-MM is *not* at the perfect lens condition (seen where $\rho$ peaks), but rather at a different frequency and complex permeability. At the perfect lens condition, the efficiency enhancement is quite low in this particular experimental prototype due to the Fabry-Perot resonances, which are clearly seen in each MM in the oscillatory of gain with frequency (and wavelength). It is important to note that the lower performance of the XYZ-MM is not due to the specific prototype considered, as the analysis for a infinite disc in Fig. 4 showed theoretically that the XYZ-MM performs lower than either the Z-M or XY-MM, just as seen in the finite disc simulation (Fig. 5, red) and the experimental prototypes (Fig. 5, cross hatched lines).

$G_\rho$ represents the efficiency enhancement considering all parameters and phenomena. As expected, $G_\rho$ tracks with $G_{max}$ for each MM configuration, Figure 5m–o. The Z-MM offers the greatest enhancement in system gain, a factor of 2.5 for the experimental case and over 3 for the simulated case. Figure 5l shows the maximum efficiency ($G_{Max}$) versus frequency. The XY-MM is the next best configuration, achieving an enhancement factor of 2.3 for the experimental case and over 3 for the simulated case. Figure 5m shows the maximum efficiency ($G_{Max}$) versus frequency This is an important result as it shows that the isotropic, XYZ-MM, is not the optimal choice. This result also demonstrates why $G_{\rho }$ is the preferred metric for MM enhanced WPT and not $\rho$ as used in some previous works^24^.

The the results demonstrated how the optimal choice of MM and optimal operating point can be determined through $G_{\rho }$. Insight into the loss mechanisms can be understood through the model proposed in Fig. 2, where considering matched loss term is key to understand loss. The focus on $G_{\rho }$ is a new and important emphasis compared to previous works that focused on $\rho$ as this $\rho$ does not account for the important losses in MM, especially from MSW.

## Methods

We developed two simulation models: an infinite axisymmetric disc and a finite axisymmetric disc, and one rectangle experimental prototype. The simulation geometry was created to match the experimental prototype, including the MM thickness, coil location and permeability (the permeability for simulation was taken from the experimental extracted permeabilities). The simulation geometry (axisymmetric) differs form the experimental only due to practical limitation of our simulations, which were much easier with an axisymmetric geometry.

### Metamaterial permeability and configurations

The MM is represented in simulation as a continuous slab with a relative permeability defined for each axis direction using Cartesian coordinates, i.e., $\mu = \mu _o [\mu _x, \mu _y, \mu _z]$. Three unique MM configurations are explored in this work, each of which is named for the direction(s) that have a non-unity relative permeability. These MM configurations represent three unique design options with individual responses and WPT-enhancing properties. Evaluating each individually will provide insight into which MM configuration proves to be the best design choice for WPT systems.

The first MM configuration, called “XY MM,” has a permeability of the format $\mu = \mu _o [\mu _x = \mu _r, \mu _y = \mu _r, \mu _z = 1]$, which indicates that the MM is “visible” to X- and Y-directed magnetic field vectors but is “transparent” to those in the Z direction. Figure 6c shows the orientation of the X, Y and Z with respect to the metamaterial. The second MM configuration is called “Z MM” and is the opposite case of the XY configuration, with $\mu = \mu _o [\mu _x = 1, \mu _y = 1, \mu _z = \mu _r]$. Both XY and Z MM configurations are anisotropic. The final MM configuration is isotropic, or “XYZ MM,” and has the same permeability for each direction: $\mu = \mu _o [\mu _x = \mu _r, \mu _y = \mu _r, \mu _z = \mu _r]$. The non-unity permeability terms in all three MM configurations are always the same value, $\mu _r$, whether they are $\mu _x$, $\mu _y$, or $\mu _z$ for any given configuration. The MM were simulated in two ways. The first is that the $\mu _r$ was varied directly and the frequency kept constant. In the second the permeability terms are a function of frequency, as shown in Fig. 5. COMSOL Multiphysics^25^ was used for all simulations.

### Metamaterial simulation—setup

A 2D axisymmetric model was used to simulate the MM, in which a 2D cross-section of the system is drawn and revolved around a central axis (*z*) to produce the equivalent of a 3D result. An axisymmetric model was chosen for its computational efficiency and because in our experience it is nearly as accurate as a more time-consuming full-3D model. The geometry of the simulation space is shown in Fig. 6, including the source and receiver coils. The MM is 2.20 inches (5.59 cm) thick. The coils are placed 4.88 inches (12.4 cm) apart, measured center-to-center. A distance of 1.34 inches (3.40 cm) separates the MM surface and the center of each coil. There are two simulations and one experimental configuration considered. The first simulation is an infinite axisymmetric cylinder (cylindrical due to the axisymmetric rotation), Fig. 6a, and is used for simulations in Fig. 3. The second configuration is shown in Fig. 6b, which is the same cylinder, however the boundary conditions at the edge are removed so that it acts as a finite geometry and will reflect MSW due to the discontinuity between the MM and air at the boundary; this creates Fabry-Perot resonances^11^, which cause constructive and destructive interference in the field enhancement of the MM. The simulation space is a square area, 39.4 inches (100 cm) per side; the outer edge of the simulation space is bounded by a matching layer.

### Permeability calculations and extraction

**Figure 7:**
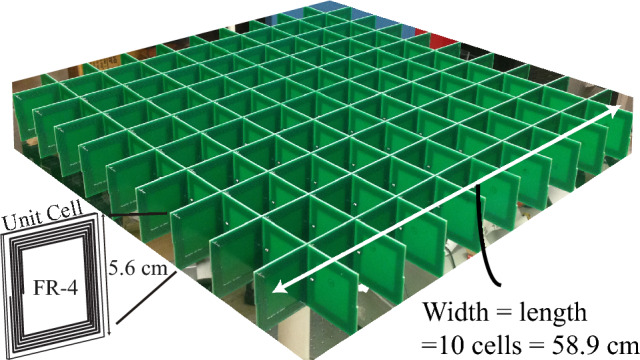
Picture of MM used in these experiments.

An analytical expression^23^ was used to match the experimentally measured complex $\mu _r$ to the deisgn parameters of a resonant *LC* MM unit cell (see inset, Fig. 7). It has been shown that one could calculate analytically the complex $\mu _r$ using the following equation:

8 $$\begin{aligned} \mu \approx 1 + \frac{F\omega ^2}{\left( \omega _o^2-\omega ^2+j\omega \omega _o/Q_{cell}\right) }, \end{aligned}$$

where *Q*, $\omega _o$ and *F* are parameters of the MM. Typically these would be derived from experimental results, however one can assume reasonable values and then update the simulations once a MM is fabricated and $\mu _r$ is measured. *Q* is the effective quality factor of the MM elements; *F* is the filling factor (typically $\approx 0.35$) and $\omega _o$ is the resonant frequency of the MM element, above which $\mu _r$ takes on negative values. For our example, we will use $Q=50$, $F=0.35$ and $\omega _o=2\pi \cdot 2.425$ MHz. These are based on the experimental MM discussed in this work; however, these numbers are reasonable starting points for any MM analysis.

In addition to ohmic losses in MM electrical components, an important and significant loss mechanism also exists in MM: Magneto-quasistatic waves (MSW). MSW can occur in a layered structure where the center material has a different permeability than the two outer layers, e.g. $\mu _r$ and $\mu _r'$ (air), Fig. 4. In the context of this work, the MM has a permeability $\mu _r$ that is different than the surrounding air ($\mu _r'=1$).

### Calculation of magnetostatic waves

Two types of magneto-quasistatic waves can occur: magnetostatic volume waves (MSV), Fig. 4a,b, when $\mu _{xx,yy}$ and $\mu _{zz}$ have different signs and magnetostatic surface waves (MSW), Fig. 4c,d, when $\mu _{xx,yy}$ and $\mu _{zz}$ are the same sign^22^. The solution for each wave can be found from boundary conditions and the dispersion relations are given below^22^:

9 $$\begin{aligned} k_{x}= & {} \pm \frac{1}{2d}\sqrt{\frac{\mu _{zz}}{\mu _{xx}}}\text {ln}\left[ \frac{1+\mu _{zz}\sqrt{\mu _{xx}/\mu _{zz}}}{1-\mu _{zz}\sqrt{\mu _{xx}/\mu _{zz}}}\right] \text{ MSW, } \end{aligned}$$

10 $$\begin{aligned}{} & {} \text {tan}\left[ \frac{ k_{x} d}{2}\sqrt{\frac{-\mu _{xx}}{\mu _{zz}}}-\frac{m\pi }{2}\right] = \frac{1}{\sqrt{-\mu _{xx}\mu _{zz}}} \text{ MVW, } \end{aligned}$$

where $m=0, 1, 2 \ldots$ is the order of the wave for MVW.

### Metamaterial design and fabrication

The realization of experimental MM for magnetostatic (magnetic field only) WPT applications has been previously reported^11,23^. Here we briefly review the methodology used for completeness. The MM is composed of a 2-D periodic array (anisotropic case) of resonant coils. The permeability of the MM can be estimated from the *Q* of the single cell and the spacing of the system using Eq. (8). The unit cell geometry is 5. cm square. A planar spiral inductor on one PCB layer was used as the inductor and a lumped 1.8 nF capacitor attached to complete the *LC* resonator with resonance at 2.4 MHz. The simulated *Q* of the cell, ignoring the loss of the capacitor, was 100. The fill factor was $F=.35$.

We experimentally characterized the permeability of each MM (Z, XY, and XYZ) using the retrieval method described in^26^. The XY-MM is shown in Fig. 7. The complex permeability is given in Fig. 5a,f,k for both real and imaginary components of the permeability. We determined that our composite MM had a resonant frequency around 2.425 MHz, with bulk *Q*-factor of approximately 50. The difference between the bulk Q and cell Q-factors (we mentioned this before as $Q_{cell} = 100$) is due to the interaction of the MM cells in the composite MM. The experimentally obtained complex permeability was used for the finite disc simulations in order to match them to the experimental results from those same MM from which the permeabilities were extracted.

## Data Availability

All data generated or analysed during this study are included in this published article and its supplementary information files.
